# Leptin stimulation of cell cycle and inhibition of apoptosis gene and protein expression in OVCAR-3 ovarian cancer cells

**DOI:** 10.1007/s12020-012-9788-7

**Published:** 2012-09-12

**Authors:** Anna Ptak, Elzbieta Kolaczkowska, Ewa L. Gregoraszczuk

**Affiliations:** 1 Department of Physiology and Toxicology of Reproduction, Chair of Animal Physiology, Jagiellonian University, Gronostajowa 9, 30-387 Krakow, Poland; 2 Department of Evolutionary Immunology, Institute of Zoology, Jagiellonian University, Gronostajowa 9, 30-387 Krakow, Poland

**Keywords:** Leptin, Apoptosis, Cell cycle, OVCAR-3 cell

## Abstract

The OVCAR-3 cell line expressing the long (ObRb) and short (ObRt) isoforms of leptin receptor mRNA was used to analyze the effect of leptin on the expression of selected genes and proteins involved in the cell cycle and apoptosis. OVCAR-3 cells were exposed to 2, 20, 40, and 100 ng/ml of leptin. Cell proliferation was determined using the alamarBlue cell viability test and flow cytometry. Apoptosis was measured using a cellular DNA fragmentation ELISA kit. The expression of selected cell cycle and apoptosis genes was evaluated by real-time PCR and confirmed by western blot. The stimulatory action of leptin on cell proliferation was observed as an increase in cells in the S and G2/M phases. Up-regulation of genes responsible for inducing cell proliferation and suppression of genes responsible for inhibition of proliferation were noted. Western blots revealed increased expression of cyclins D and A and inhibition of p21WAF1/CIP1 protein expression by leptin. Inhibition of DNA fragmentation was observed under all leptin doses. Suppression of genes involved in the extrinsic and intrinsic apoptotic pathway was observed. Western blots illustrated decreased Bad, TNFR1, and caspase 6 protein expression in response to leptin treatment. Leptin promotes ovarian cancer cell line growth by up-regulating genes and proteins responsible for inducing cell proliferation as well as down-regulating pro-apoptotic genes and proteins in apoptotic pathways. Results of this study warrant examining the relationship between the risk of ovarian cancer and elevated leptin levels in obese women.

## Introduction

Leptin is a multifunctional peptide hormone with wide-ranging biologic activities, including appetite regulation, bone formation, reproductive function, and angiogenesis. Circulating levels of leptin are strongly correlated to body fat content and are markedly elevated in obese compared with normal-weight individuals [1, 2]. Plasma leptin levels have been reported to be higher in overweight and obese women (37.7 ng/ml) than in normal-weight women (3.92–16.9 ng/ml) [2–4]. In some obese individuals, leptin levels can reach 100 ng/ml [5]. Interestingly, recent studies have demonstrated that leptin stimulates growth, migration, invasion, and angiogenesis in tumor cell models, suggesting that it can promote an aggressive cancer phenotype [6, 7]. Epidemiological studies have suggested a positive correlation between obesity and increased risk of different cancers, including ovarian cancer [8–10]. Several studies have addressed the possible role of leptin, the product of the obesity gene (*Ob*), in ovarian cancer development and progression [11–13]. A recent study by Uddin et al. [13] showed a significant association between leptin receptor (Ob-R) overexpression and poor progression-free survival in 59.2 % of epithelial ovarian cancers. In vitro studies have demonstrated that leptin induces proliferation in the ovarian cancer cell line BG-1 [11, 12], and it has been shown to inhibit apoptosis in ovarian epithelial cancer cell lines SKOV3 and MDAH2774 [13]. Accumulating evidence indicates that leptin has an effect on ovarian cancer growth, but information on its molecular mechanism with respect to regulation of the cell cycle and apoptosis is limited.

Based on the data of Choi et al. [11], who characterized both long (ObRb) and short (ObRt) isoforms of leptin mRNA, as well as our previous study [14] showing protein expression of both isoforms of the leptin receptor in the immortalized OVCAR-3 ovarian cancer cell line, we used this cell line to analyze the effect of leptin on the expression of selected genes involved in the cell cycle and apoptosis.

## Materials and methods

### Reagents

RPMI 1640 medium without phenol red, fetal bovine serum (FBS, heat-inactivated), penicillin, streptomycin, and trypsin EDTA were obtained from PAA Laboratories GmbH (Colbe, Germany). Human leptin, bovine serum albumin (BSA), and trypan blue were obtained from Sigma Chemical Co., (St. Louis, MO, USA).

### Cell culture and treatments

The human ovarian epithelial carcinoma cell line OVCAR-3 was obtained from the American Type Culture Collection (Manassas, VA, USA). The cells were routinely cultured in RPMI 1640 medium without phenol red supplemented with 50 U/ml penicillin, 50 μg/ml streptomycin, and 10 % v/v heat-inactivated FBS in a humidified incubator with 5 % CO_2_ at 37 °C. The cells were switched to medium without serum 24 h before each experiment.

### Cell proliferation

Cell proliferation was measured using the alamarBlue cell viability reagent (Invitrogen, Paisley, UK) according to the manufacturer’s instructions. The cells were seeded in 96-well culture plates at a density of 1.5 × 10^4^ cell/well and then incubated in RPMI 1640 supplemented with 5 % FBS as a control medium or with four different doses (2, 20, 40, and 100 ng/ml) of leptin for 24, 48, and 72 h. The medium was changed daily, adding new medium and new test compounds. The alamarBlue stock solution was aseptically added to the wells after 24, 48, and 72 h of culture in amounts equal to 10 % of the incubation volume. Resazurin reduction was determined after 4 h of incubation by measuring the fluorescence at 560 nm (excitation)/590 nm (emission) using an FLx800 fluorescence microplate reader (BioTek, Winooski, VT, USA).

### Flow cytometry analyses

The cells were seeded in 6-well culture plates at a density of 0.5 × 10^6^ cell/well in RPMI 1640 supplemented with 5 % FBS as a control medium or with 40 ng/ml of leptin for 24 and 48 h. For analysis of cell proliferation, cell samples were stained with propidium iodide (PI). Briefly, after centrifugation (10 min at 1,500 rpm, 4 °C), the cell pellets were adjusted to 1 × 10^6^ cell/ml and re-suspended in the PI staining buffer. The buffer contained 7.5 × 10^−5^ M PI, 10 mM Tris base, 10 mM NaCl, 700 U/l RNase, 0.1 ml/100 ml Nonidet P-40 (all from Sigma-Aldrich Chemical Co., St. Louis, MO, USA) and was adjusted to pH 8.0. The re-suspended cell pellets were kept in the buffer for 30 min in the dark at 4 °C. Samples were then analyzed in a FACScalibur flow cytometer (Becton-Dickinson, San Jose, CA, USA) to assess the cell cycle DNA profile. In total, 20,000 threshold events per sample were collected and analyzed based on their FL2 fluorescence (emission at 585 nm, excitation at 488 nm). The resulting data were analyzed using CellQuest Pro software (Becton-Dickinson). Percentages of cells present in the various phases of the cell cycle were calculated using Cell Quest by gating for the following cell populations: A (apoptotic cells; <2*n*), G1/G0 [cells in gap 1 (G1) and entering into the quiescent phase (G0); 2*n*], S (cells in the DNA synthesis phase; >2*n*), and G2 + M [cells in gap 2 (G2; 4*n*), and entering into the mitotic phase (M; <4*n*)].

### Bromodeoxyuridine (BrdU) incorporation assay

DNA fragmentation was determined using the Cellular DNA Fragmentation ELISA Kit (Roche Applied Science, Mannheim, Germany). This assay is based on the quantitative detection of BrdU-labeled DNA fragments. After exposure to BrdU for 18 h, the cells were re-seeded in 96-well culture plates at 1.5 × 10^4^ cell/well and then incubated in RPMI 1640 as a control medium or with leptin at concentrations of 2, 20, 40, and 100 ng/ml. After 24 h, DNA fragmentation was determined according to the manufacturer’s instructions. Absorbance values were measured spectrophotometrically at 450 nm using an ELx808 ELISA reader (BioTek).

### Real-time PCR analysis

Cells were seeded in 6-well culture plates at 1 × 10^6^ cell/well in RPMI 1640 supplemented with 5 % FBS (for cell cycle analysis) or without serum (for apoptosis analysis) as the control medium, or with 40 ng/ml of leptin for 24 h. The choice of this dose was based on previous cell proliferation experiments. Total RNA from OVCAR-3 cells was isolated using the High Pure RNA Isolation Kit (Roche Applied Science). RNA purity and quantity were determined spectrophotometrically according to optical densities at 260 and 280 nm. cDNA synthesis was performed using the Transcriptor First Strand cDNA Synthesis Kit (Roche Applied Science) with a mixture of anchored-oligo(dT)_18_ primers and random hexamer primers according to the manufacturer’s protocol. Amplifications were performed in duplicate using the LightCycler 480 System (Roche Applied Science) and the Real-Time Ready Human Apoptosis Panel and Real-Time Ready Human Cell Cycle Regulation Panel (Roche Applied Science) in combination with the LightCycler 480 Probes Master (Roche Applied Science) according to the manufacturer’s instructions. PCR was performed in a final volume of 20 μl, including 50 ng cDNA per reaction. The PCR conditions were as follows: pre-incubation for 10 min at 95 °C, amplification for 45 cycles (10 s at 95 °C, 30 s at 60 °C, and 1 s at 72 °C). A positive control (to check for RNA degradation) and negative control (to detect residual genomic DNA) were run simultaneously with every assay according to the manufacturer’s protocol. The relative expression of the analyzed genes was normalized to seven reference genes in the panel using the E-Method from Roche Applied Science, which analyzes the amplification efficiency of target and reference genes using relative standards. These standards are serial dilutions of a single sample (for example, undiluted, 1:10, 1:100, and so on), where the concentration is expressed in relative units (for example, 1, 0.1, 0.01, and so on). Using dilutions to generate a standard curve, the E-Method avoids the time-consuming preparation of artificial or cloned standards and the determination of their absolute values [15].

### Western blot analysis

The cells were seeded in 24-well culture plates at 1 × 10^5^ cell/well in RPMI 1640 supplemented with 5 % FBS (for cell cycle analysis) or without serum (for apoptosis analysis) as the control medium, or with leptin at 40 ng/ml for 24 and 48 h. The cells were washed with ice-cold PBS and lysed in ice-cold buffer. The protein concentration of the cell lysate was determined by Bradford reagent (Bio-Rad Protein Assay, Bio-Rad Laboratories, Munchen, Germany). Protein (30 µg from each treatment group) was separated by 10 and 15 % SDS-PAGE and transferred to PVDF membranes using a Bio-Rad Mini-Protean 3 apparatus (Bio-Rad Laboratories). The blots were blocked for 2 h with 5 % w/v BSA and 0.1 % v/v Tween 20 in 0.02 M Tris Buffered Saline buffer (TBS). The blots were incubated overnight at 4 °C with antibodies specific for cyclin D1, cyclin A, p21 Waf1/Cip1, TNFR1, caspase 6, and Bad from Cell Signaling Technology (Danvers, MA, USA). Loading controls consisted of immunoblotting the same membranes with β-actin antibody (Sigma Chemical Co.,). After incubation with the primary antibody, the membranes were washed three times and incubated for 1 h with a horseradish peroxidase-conjugated secondary antibody from Cell Signaling Technology for cyclin D1, cyclin A, p21 Waf1/Cip1, TNFR1, caspase 6, and Bad (from DakoCytomation, Glostrup, Denmark) for β-actin. Immunopositive bands were visualized using Western Blotting Luminol Reagents (Santa Cruz Biotechnology Inc., Santa Cruz, CA, USA) and quantified using densitometry analysis (EasyDens, Cortex Nowa, Poland).

### Statistical analysis

Data were expressed as mean ± SEM from four independent experiments performed in triplicate. Statistical analyses were performed using GraphPad Prism 5. Data were analyzed by one-way analysis of variance (ANOVA) followed by Tukey’s honestly significant difference (HSD) multiple range test. A value of *P* < 0.05 was considered statistically significant.

## Results

### Cell proliferation (alamarBlue assay)

**Fig. 1:**
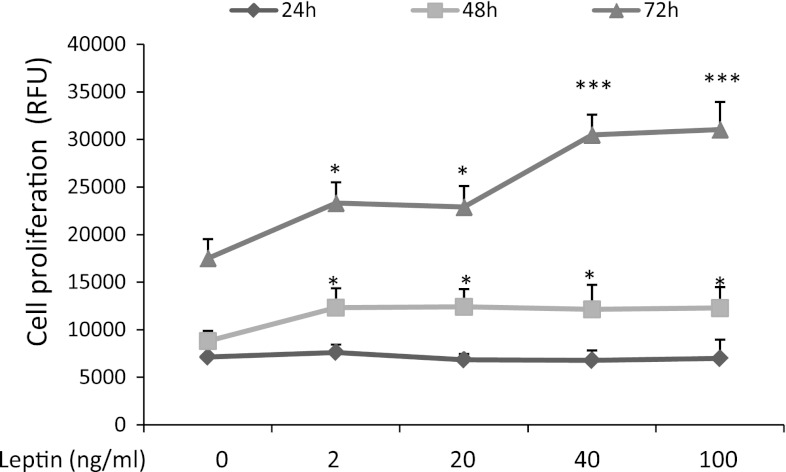
Effects of leptin on OVCAR-3 cell proliferation. The cells were treated with leptin at 2, 20, 40, and 100 ng/ml for 24, 48, and 72 h and then analyzed using the alamarBlue assay. Values are mean ± SEM. *^,^***Significantly different from control at *P* < 0.05 and *P* < 0.001, respectively

Growth of OVCAR-3 cells in response to various doses of leptin was tested for 24, 48, and 72 h. An increase in cell proliferation was noted under all leptin doses at 48 h of treatment (1.3-fold; *P* < 0.05; Fig. 1), with 2 and 20 ng/ml of leptin at 72 h of treatment (1.3-fold; *P* < 0.05; Fig. 1), and with 40 and 100 ng/ml of leptin at 72 h of treatment (1.6-fold; *P* < 0.001; Fig. 1). The sub-maximal leptin dose (40 ng/ml) was chosen for subsequent experiments.

### Distribution in the cell cycle (flow cytometry)

**Fig. 2:**
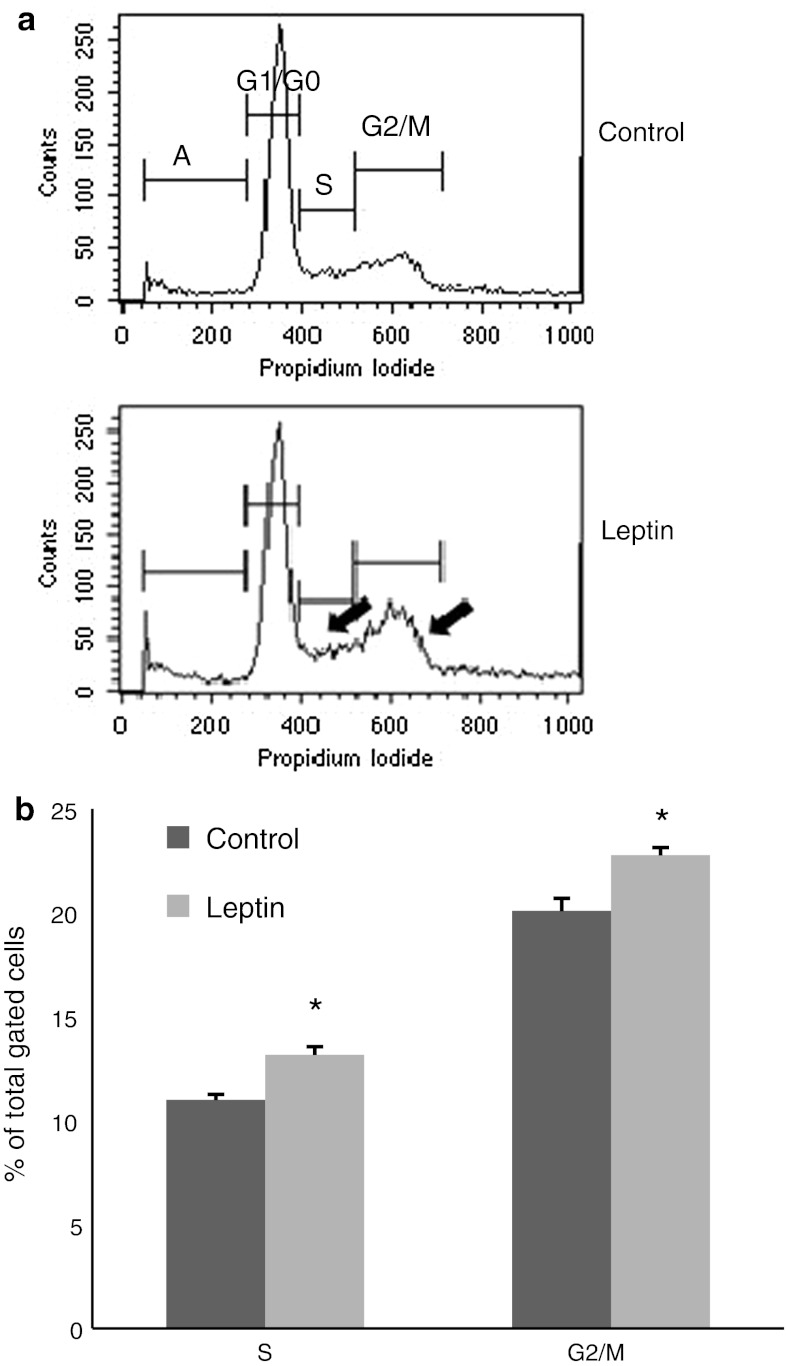
Effects of leptin (40 ng/ml) on cellular DNA content and cell proliferation. **a** Representative histograms as estimated by flow cytometry after propidium iodide staining. *G0/G1* resting phase, in preparation for S; *S* DNA synthesis phase; *G2* *+* *M* cells in gap 2 and entering into the mitotic phase; *A* apoptotic cells. *Arrows* indicate peaks of S and G2 + M cells. **b** Relative percentages of the cell population in S and G2/M phase are presented in the total gated cell numbers. Values are mean ± SEM. *Significantly different from the control at *P* < 0.05

Figure 2 presents the distribution of cells in the various phases of the cell cycle. In the controls, most OVCAR-3 cells were in the G0/G1 phase at any given point of time. At 48 h, significantly different percentages of cells were in the S phase (DNA content 2–4*n*; 13.16 ± 0.40 % for leptin-treated cells vs. 10.99 ± 0.28 % for the control) and G2 + M phase (4*n*; 22.76 ± 0.38 for leptin-treated cells vs. 20.08 ± 0.62 % for the control) (*P* < 0.05; Fig. 2a, b).

### Cell cycle gene expression analysis

A 1.2- to 1.9-fold induction of G1/S-specific cyclin D1 (*CCND1*), G2/mitosis-specific cyclin A2 (*CCNA2*), transcription factors E2F3 (*E2F3*), and proliferating cell nuclear antigen (*PCNA*) (all genes inducing cell proliferation) was measured in cells exposed to leptin. In parallel, 1.9- and 1.2-fold suppressions of cyclin-dependent kinase inhibitor p21 (*P21CIP1*) and p27 (*P27KIP1*), respectively, and a 1.2-fold suppression of growth arrest and DNA-damage-inducible protein GADD45 alpha (*GADD45A*) (genes inhibiting proliferation) were observed. No change was found in the levels of cell division protein kinases 1 (*CDK1*), 4 (*CDK4*), and 6 (*CDK6*), wee1-like protein kinase (*WEE1*), or transcription factors E2F1 (*E2F1*) or E2F2 (*E2F2*). We also noted no change in the levels of the S-phase regulatory protein cyclin E (*CCNE2, CCNE2*) or its partner protein CDK2 (*CDK2*), or in G2/mitotic-specific cyclin B1 (*CCNB1*) (*P* < 0.05, *P* < 0.001; Table 1).

**Table 1:** Changes in selected cell cycle genes’ expression in OVCAR-3 cells exposed to 40 ng/ml leptin for 24 h

Assay ID	Transcript	Description	Gene symbol	Average fold change
G1/S phase				
100844	ENST00000227507	G1/S-specific cyclin-D1 (PRAD1 oncogene)	*CCND1*	1.3*
102813	ENST00000372991	G1/S-specific cyclin-D3	*CCND3*	1.0
101418	ENST00000257904	Cell division protein kinase 4	*CDK4*	1.0
101427	ENST00000265734	Cell division protein kinase 6	*CDK6*	1.0
100848	ENST00000262643	G1/S-specific cyclin-E1	*CCNE1*	1.0
100850	ENST00000308108	G1/S-specific cyclin-E2	*CCNE2*	1.0
101416	ENST00000266970	Cell division protein kinase 2	*CDK2*	1.0
101538	ENST00000267163	Retinoblastoma-associated protein (pRb)	*RB1*	1.0
102827	ENST00000343380	Transcription factor E2F1 (E2F-1)	*E2F1*	1.0
102830	ENST00000361729	Transcription factor E2F2 (E2F-2)	*E2F2*	1.0
102834	ENST00000346618	Transcription factor E2F3 (E2F-3)	*E2F3*	1.2*
101524	ENST00000379160	Proliferating cell nuclear antigen (PCNA)	*PCNA*	1.2*
G2/M phase				
102811	ENST00000274026	Cyclin-A2 (cyclin-A)	*CCNA2*	1.3*
101373	ENST00000256442	G2/mitotic-specific cyclin-B1	*CCNB1*	1.1
101376	ENST00000288207	G2/mitotic-specific cyclin-B2	*CCNB2*	1.0
101406	ENST00000293968	Cell division control protein 2 homolog	*CDK1*	1.0
102820	ENST00000302506	M-phase inducer phosphatase 1	*CDC25A*	1.0
102823	ENST00000245960	M-phase inducer phosphatase 2	*CDC25B*	1.15
102826	ENST00000323760	M-phase inducer phosphatase 3	*CDC25C*	1.0
102849	ENST00000299613	Wee1-like protein kinase	*WEE1*	1.0
Cell cycle progresion inhibitors				
102909	ENST00000244741	Cyclin-dependent kinase inhibitor 1 (p21)	*P21CIP1*	−1.9**
100855	ENST00000228872	Cyclin-dependent kinase inhibitor 1B (cyclin-dependent kinase inhibitor p27)	*P27KIP1*	−1.2*
101471	ENST00000370986	Growth arrest and DNA-damage-inducible protein GADD45 alpha (DNA-damage-inducible transcript 1)	*GADD45A*	−1.2*

Values are mean ± SEM. *^,^** Significantly different from control at *P* < 0.5 and *P* < 0.001, respectively. Control value = 1.0

### Cell cycle protein expression analysis

**Fig. 3:**
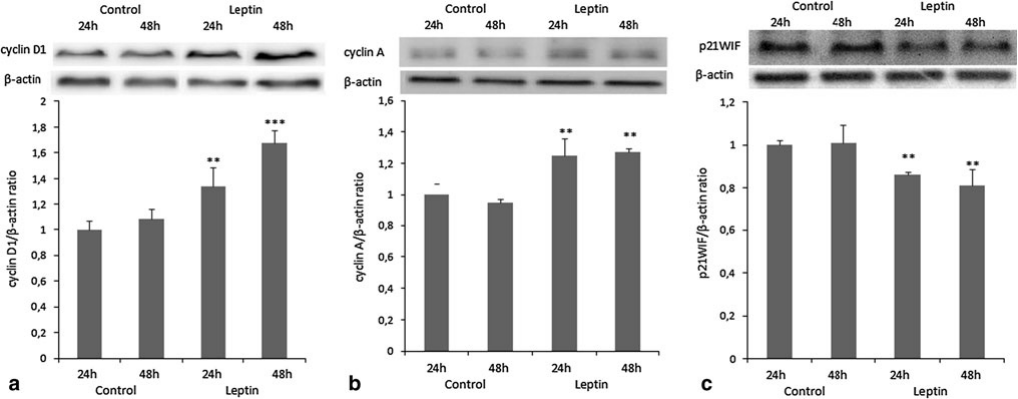
Effects of leptin (40 ng/ml) on **a** cyclin D1 (36 kDa), **b** cyclin A (55 kDa), and **c** p21WAF1/CIP1 (21 kDa) protein expression in OVCAR-3 cells. Cells were treated with leptin for 24 and 48 h. β-actin (42 kDa) served as the loading control, and densitometry values of cyclin D1, cyclin A, and p21WAF1/CIP1 bands were normalized to β-actin. Signal intensities are expressed in arbitrary units. The values are mean ± SEM. **^,^***Significantly different from control at *P* < 0.01 and *P* < 0.001, respectively

The protein expression of *CCND1*, *CCNA2*, and *P21CIP1*, the most changed genes during the cell cycle, was analyzed by western blot. As with the genes’ expression, an increase in cyclin D1 (an S-phase-associated protein) and cyclin A2 (a G2/M-phase-associated protein) and a decrease in p21WAF1/CIP1 (inhibitor of S-phase-associated protein) expression following leptin treatment were noted (*P* < 0.01, *P* < 0.001; Fig. 3a–c).

### Apoptosis analysis by DNA fragmentation

**Fig. 4:**
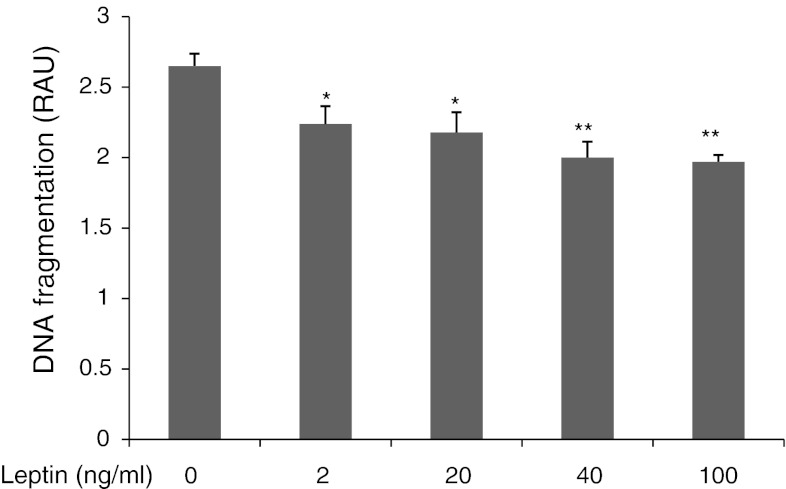
Effects of leptin on DNA fragmentation in OVCAR-3 cells. Cells were treated with leptin at 2, 20, 40, and 100 ng/ml for 24 h and then analyzed with a Cellular DNA Fragmentation ELISA kit. RAU, relative absorbance units. Values are mean ± S.E.M. *^,^**Significantly different from control at *P* < 0.05 and *P* < 0.01, respectively

The statistically significant inhibition of DNA fragmentation to 84, 82, 75, and 74 % of control values was observed with leptin treatments of 2, 20, 40, and 100 ng/ml, respectively (*P* < 0.05, *P* < 0.01; Fig. 4).

### Apoptotic gene expression analysis

As shown in Table 2, leptin had no effect on *FAS*, *CRADD*, caspase 2 (*CASP2*), or caspase 8 (*CASP8*) mRNA levels, whereas we observed 1.4- to 1.8-fold suppression of *TNFR1*, *FADD*, and caspase 10 (*CASP10*). These results suggest a lack of activation of the Fas-dependent apoptotic pathway and inhibition of the TNFR apoptotic pathways. All of these genes are involved in the extrinsic apoptotic pathway. A 1.2-fold suppression of caspase 9 (*CASP9*) and 2.0-fold suppression of the gene expression of its partner protein Apaf-1 (*APAF1*) were noted. In addition, we measured a range of 1.3- to 2.2-fold suppression of caspases 3 (*CASP3*), 6 (*CASP6*), and 7 (*CASP7*), which are well-known effector caspases, and *CAD* and *ICAD*, which are responsible for DNA fragmentation (all of these genes are involved in the intrinsic apoptotic pathway). Leptin had no effect on *BCL2*, *BCLW*, *BCLX*, or *MCL1* levels (pro-survival genes), whereas a range of 1.2- to 2.1-fold suppression of *BAD*, *BAX*, *BAK1*, *BID*, and *BOK* (pro-apoptotic genes) was observed. We also noted no change in the levels of the endonuclease G (*ENDOG*) genes involved in CAD-independent DNA fragmentation or in the tumor-suppressor gene p53 (*TP53*) (*P* < 0.05, *P* < 0.01, *P* < 0.001; Table 2).

**Table 2:** Changes in selected apoptotic genes’ expression in OVCAR-3 cells following exposure to 40 ng/ml leptin for 24 h

Assay ID	Transcript	Description	Gene symbol	Average fold change
Extrinsic pathway				
102679	ENST00000162749	Tumor necrosis factor receptor superfamily member 1A precursor (p60)	*TNFR1*	−1.8***
100426	ENST00000371875	Tumor necrosis factor receptor superfamily member 6 precursor (FASLG receptor)	*FAS*	−1.1
100417	ENST00000301838	Protein FADD (FAS-associated death domain protein)	*FADD*	−1.54**
100289	ENST00000332896	Death domain-containing protein CRADD (caspase and RIP adapter with death domain)	*CRADD*	1.0
100227	ENST00000358485	Caspase-8 precursor	*CASP8*	−1.1
100204	ENST00000272879	Caspase-10 precursor	*CASP10*	−1.45**
100210	ENST00000310447	Caspase-2 precursor	*CASP2*	1.0
Intrinsic pathway				
Pro-survival				
100083	ENST00000398117	Apoptosis regulator Bcl-2	*BCL2*	1.0
104039	ENST00000250405	Apoptosis regulator Bcl-W (Bcl-2-like 2 protein)	*BCLW*	1.0
100088	ENST00000307677	Apoptosis regulator Bcl-X (Bcl-2-like 1 protein)	*BCLX*	1.0
102930	ENST00000369026	Induced myeloid leukemia cell differentiation protein Mcl-1 (Bcl-2-related protein EAT/mcl1)	*MCL1*	1.0
Pro-apoptotic				
104034	ENST00000394532	Bcl2 antagonist of cell death (BAD)	*BAD*	−2.1***
100068	ENST00000374467	Bcl-2 homologous antagonist/killer (apoptosis regulator BAK)	*BAK1*	−1.5**
102861	ENST00000293288	Apoptosis regulator BAX	*BAX*	−1.3*
100122	ENST00000342111	BH3-interacting domain death agonist (BID)	*BID*	−1.2*
100126	ENST00000216115	Bcl-2-interacting killer (Apoptosis inducer NBK)	*BIK*	1.0
100165	ENST00000318407	Bcl-2-related ovarian killer protein (Hbok)	*BOK*	−1.8***
100233	ENST00000333868	Caspase-9 precursor	*CASP9*	−1.2*
102892	ENST00000333991	Apoptotic protease-activating factor 1 (Apaf-1)	*APAF1*	−1.8***
100213	ENST00000308394	Caspase-3 precursor	*CASP3*	−1.3*
100222	ENST00000265164	Caspase-6 precursor	*CASP6*	−2.0***
100224	ENST00000369331	Caspase-7 precursor	*CASP7*	−1.5**
102620	ENST00000264705	CAD protein	*CAD*	−1.25*
102630	ENST00000377038	DNA fragmentation factor subunit alpha (DNA fragmentation factor 45 kDa subunit)	*ICAD*	−1.25*
Caspase-independent pathway				
102632	ENST00000372642	Endonuclease G, mitochondrial precursor	*ENDOG*	1.0
101277	ENST00000396473	Cellular tumor antigen p53 (Tumor suppressor p53)	*TP53*	1.0

Values are mean ± SEM. *^,^**^,^*** Significantly different from control at *P* < 0.05, *P* < 0.01, and *P* < 0.001, respectively. Control value = 1.0

### Apoptotic protein expression analysis

**Fig. 5:**
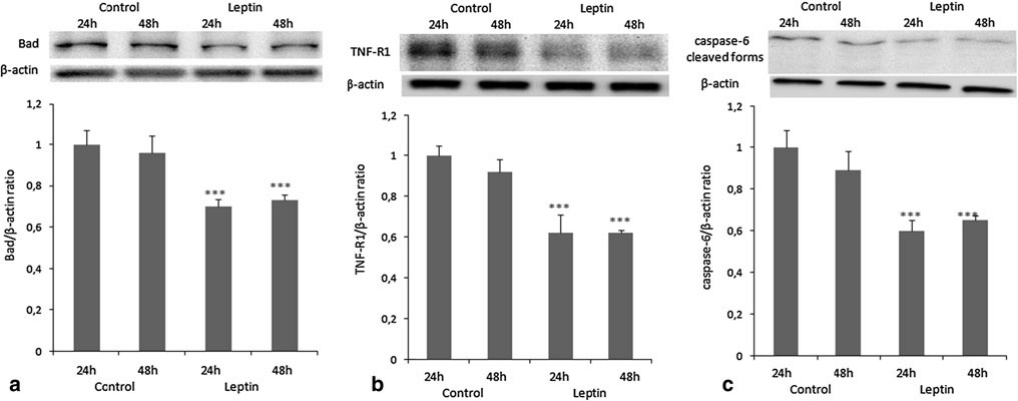
Effects of leptin (40 ng/ml) on **a** Bad (23 kDa), **b** TNFR1 (55 kDa), and **c** caspase 6 (35, 15 kDa) protein expression in OVCAR-3 cells. Cells were treated with leptin for 24 and 48 h. β-actin (42 kDa) served as the loading control, and densitometry of TNFR1, Bad, and caspase 6 bands were normalized to β-actin. Signal intensities are expressed in arbitrary units. Values are mean ± SEM. ***Significantly different from control at *P* < 0.001

The protein expression of *BAD*, *TNFR1*, and *CASP6*, the apoptotic genes that showed the largest changes in gene expression level, was analyzed by western blot. Leptin-induced inhibition of Bad (a component of the intrinsic pathway and a pro-apoptotic Bcl-2 family protein; *P* < 0.001; Fig. 5b), TNFR1 (a component of the extrinsic pathway, tumor necrosis factor receptor 1; *P* < 0.001; Fig. 5a), and caspase 6 protein (executor caspases; *P* < 0.001; Fig. 5c) expression was noted. The cleaved forms of caspase 6 were almost undetectable following leptin treatment.

## Discussion

We evaluated the effects of leptin on cell proliferation and apoptosis in the human ovarian cancer cell line OVCAR-3. Because we found a dose-dependent stimulatory effect on cell proliferation and an inhibitory effect on apoptosis, we expected that the gene products involved in these processes would also be affected.

### Effects on cell proliferation

Using the alamarBlue test, which is based on the detection of cellular metabolic activity, we defined a dose-dependent (from 2 ng/ml to 100 ng/ml) stimulatory effect of leptin on OVCAR-3 cell proliferation. This observation is in agreement with Uddin et al. [13], who observed a dose-dependent (from 10 ng/ml) stimulatory effect of leptin on proliferation of the ovarian epithelial cancer cell line MDAH2774. On the other hand, Choi et al. [11] showed that leptin at 1 and 10 ng/ml doses has no effect on human ovarian cancer BG-1 cell proliferation, but stimulates cell proliferation at doses of 100 and 1,000 ng/ml.

Flow cytometry analysis showed that leptin promotes cell cycle progression, as demonstrated by an increased cell population in both the S and G2/M phases. Our results are consistent with Catalano et al. [16], who noted an increased number of Ishikawa human endometrial cancer cells in the S phase after 24 h of leptin treatment (100 and 1,000 ng/ml). In addition, Chen et al. [17] observed that 48-h treatment of ZR-75-1 breast cancer cells with leptin (100 ng/ml) increases the cell population in both S and G2/M phases.

Alterations of the mechanisms controlling cell cycle progression play a relevant role in the pathogenesis of different human neoplasias. Because the cell cycle is regulated by the coordinate action of cyclin-dependent kinases (cdks), specific cyclin proteins, and cdk inhibitors, we focused on the analysis of cell cycle-associated genes to gain insight into the mechanism of leptin’s proliferative action. Using real-time PCR, we found that leptin (40 ng/ml) impacts proliferation by up-regulating cell cycle progression genes and suppressing cell cycle inhibitor genes. We observed up-regulation of mRNAs for *CCND1*, *CCNA2*, *PCNA*, and *E2F3* (genes responsible for inducing cell proliferation) and down-regulation of mRNAs for *P21CIP1*, *P27KIP1*, and *GADD45A* (genes responsible for inhibiting proliferation). Up-regulation of *CCND1* and *CCNA2* and down-regulation of *P21CIP1*, the most changed genes, were also observed at the protein level by western blot. Cyclin D1, an important cell cycle regulator, is required for completion of the G1/S transition in normal mammalian cells [18, 19] and is one of the most commonly affected proteins in abnormal states, such as cancer. Our results are consistent with previous studies showing similar leptin induction of cyclin D1 expression in human breast cancer [16, 17, 20], the colon cancer cell line HT-29 [21], and human hepatocarcinoma cells [22]. As cells progress into the S phase, cyclin A is expressed and becomes the primary cyclin associated with cdk2. Overexpression of cyclin A has been indicated in various human tumors, including breast [23], prostate [24], and ovarian cancer [25, 26]. To the best of our knowledge, this is the first report of leptin induction of cyclin A expression in human ovarian cancer cells. In the present study, we observed suppression of p21WAF1/CIP1, which acts as a universal inhibitor of cdks, and arrests the cell cycle at both G1/S and G2/M restriction points [27]. Our results are consistent with previous studies showing a similar decrease in p21WAF1/CIP1 expression under the influence of leptin in human breast cancer cells [17] and Ishikawa human endometrial cancer cells [16]. Furthermore, low levels of p21WAF1/CIP1 have been shown to be a marker of poor overall survival in ovarian cancer patients [28]. Down-regulation or inactivation of the cdk inhibitors, including p21CIP1, p27KIP1, and p16INK4a, which normally cause G1 arrest by binding to cyclin-cdk complexes, is often observed in a variety of human tumors, rendering the cell susceptible to uncontrolled extracellular proliferation signals [29].

To summarize, we suggest that leptin may promote progression from G1 to S phase by stimulating expression of cyclin D1 and inhibiting that of p21WAF1/CIP1, as well as progression to the S phase by stimulating the expression of cyclin A in an ovarian cancer cell line.

### Action on cell apoptosis

A perturbation of the balance between cell proliferation and apoptosis has been suggested to play a significant role in mediating tumor progression. Surprisingly, data concerning the anti-apoptotic actions of leptin in ovarian cancer cells are limited. Herein, we observed that leptin inhibits OVCAR-3 cell apoptosis in a dose-dependent manner, as measured by DNA fragmentation. To our knowledge, there has been only one study addressing leptin in ovarian cancer cells [13], which showed that 100 ng/ml of leptin significantly prevents serum-starved apoptosis in the ovarian epithelial cancer cell lines SKOV-3 and MDAH2774. In previously published data, using physiologically relevant ovarian tissue, we illustrated the inhibitory action of leptin on cell apoptosis in pig ovarian follicles [30] and the developing corpus luteum [31]. There are also data showing an anti-apoptotic effect of leptin in breast cancer cell lines [7].

Cells undergo apoptosis through two major pathways: the extrinsic (death receptor) pathway and the intrinsic (mitochondrial) pathway. A hallmark of apoptosis is fragmentation of nuclear DNA. This process involves the caspase 3-dependent DNase CAD or can occur independently of caspase activity. In the present study, we found that 40 ng/ml of leptin acts by suppressing the expression of pro-apoptotic genes, such as *TNFR1*, *FADD*, *CASP3*, *6*, *7*, *9*, and *10*, *CAD*, *ICAD*, *BAX*, *BAD*, *BAK1*, *BID*, *BOK*, and *APAF1*. *TNFR1*, *BAD*, and *CASP6* were the most down-regulated genes, and these changes were also observed at the protein level, as measured by western blot. Using a gene microarray, Perera et al. [32] observed that in MCF-7 cells, leptin (500 ng/ml) induces the expression of anti-apoptotic genes *BCL2* and survivin and reduces the expression of other apoptotic genes (*TRAF*-interacting protein and *TRADD*) involved in the tumor necrosis factor (TNF)-induced apoptotic pathway. Their observation is partially consistent with our results, suggesting that leptin suppresses the TNF-induced extrinsic apoptosis pathway. Our data further showed suppression of TNFR1 at both the gene and protein expression levels. The leptin-induced reduction of Bax protein expression in T47-D breast cancer cells observed by Nkhata et al. [33], and in *BAX* gene expression observed here in OVCAR-3 cells, suggests that leptin also acts on the intrinsic apoptotic pathway. Down-regulation of TGFB1-induced Bax expression in hepatocarcinoma cells was described by Chen et al. [22]. Both apoptotic pathways lead to activation of the executioner caspases, caspase 3, 6, and 7, which are the main proteases degrading the cell. To the best of our knowledge, we are the first to show down-regulation of caspase 6 (executor caspase) at the gene and protein levels. Jiang et al. [34] showed leptin suppression of docetaxel-induced apoptosis in MCF-7 breast cancer cells via inhibition of caspase 9 activity, indirectly confirming our results. Similar to caspase 6, we observed inhibition of caspase 3 and caspase 7 mRNA expression by leptin, suggesting that leptin inhibits caspase-dependent apoptosis. Thus, we suggest that leptin prevents caspase-dependent apoptosis in OVCAR-3 cells by down-regulating pro-apoptotic proteins in both extrinsic (TNFR1) and intrinsic (BAD, caspase 6) apoptosis pathways.

In conclusion, our results are the first to demonstrate the molecular mechanism involved in the regulation of the cell cycle and apoptosis by leptin in an epithelial ovarian cancer cell line. The combination of proliferative and anti-apoptotic effects, by up-regulating genes and proteins responsible for inducing cell proliferation as well as down-regulating pro-apoptotic genes and proteins in the apoptotic pathways, clarifies the role of leptin signaling in the progression of ovarian cancer. Taking into consideration the limitations of in vitro studies, in vivo studies should be performed to confirm leptin’s contribution to ovarian cancer progression.
